# Short-term repeatability and postprandial effect assessment of liver perfusion quantification in healthy subjects using arterial spin labeling MRI

**DOI:** 10.1186/s13244-025-02051-0

**Published:** 2025-08-05

**Authors:** Pengling Ren, Xia Ma, Yawen Liu, Jinxia Zhu, Yi Sun, Bernd Kuehn, Zhenghan Yang, Penggang Qiao, Rui Wang, Zhenchang Wang

**Affiliations:** 1 Department of Radiology, Beijing Friendship Hospital, Capital Medical University, Beijing, China; 2 Department of Ultrasound, Beijing Friendship Hospital, Capital Medical University, Beijing, China; 3 Precision and Intelligence Medical Imaging Lab, Beijing Clinical Research Institute, Beijing Friendship Hospital, Capital Medical University, Beijing, China; 4 MR Research Collaboration, Siemens Healthineers, Shanghai, China; 5 Collaboration office, Siemens Healthineers, Beijing, China; 6 MR Application Predevelopment, Siemens Healthineers AG, Forchheim, Germany

**Keywords:** Liver perfusion, Magnetic resonance imaging, Arterial spin labeling, Repeatability, Postprandial effect

## Abstract

**Objectives:**

To assess the stability of liver perfusion quantification using arterial spin labeling MRI (ASL-MRI) in healthy subjects.

**Materials and methods:**

The arterial and portal venous liver perfusion were measured with two pseudo-continuous ASL acquisitions at a 3.0-Tesla MRI system. To assess the short-term repeatability of ASL-MRI, twelve healthy subjects underwent three consecutive ASL examinations in the fasting state. Following meal ingestion, the postprandial liver perfusion was measured. Changes in liver perfusion measured by ASL before and after meal ingestion, and their correspondence with portal vein hemodynamic variations assessed by Doppler ultrasonography (US), were analyzed to evaluate the stability of ASL in detecting postprandial perfusion alterations.

**Results:**

The arterial and portal venous liver perfusions in healthy volunteers under the fasting condition were 59.3 ± 17.8 and 237.6 ± 71.9 mL/100 g/min, respectively. Both the arterial and portal venous liver perfusion results demonstrated excellent short-term repeatability (ICCs, 0.97, 0.96; CVs, 6.43%, 6.17%). Furthermore, Bland–Altman plots indicated a high degree of consistency between every two pairs of the three measurements. Compared to the fasting state, the relative changes in postprandial portal venous perfusion measured by ASL-MRI demonstrated a moderate correlation (Pearson correlation coefficient *r* = 0.66) and good agreement (with all data points in the Bland–Altman plot falling within the limits of agreement) with those in portal vein blood flow measured by Doppler US.

**Conclusion:**

ASL-MRI enables reliable quantification of liver perfusion in healthy individuals under both constant conditions and altered perfusion state induced by a meal. It holds great promise as a non-invasive tool for diagnosing liver disease.

**Critical relevance statement:**

The short-term repeatability and postprandial effect of liver perfusion quantification using arterial spin labeling MRI in healthy subjects both exhibited excellent performance, indicating the potential of this technique as a non-invasive tool for diagnosing liver diseases.

**Key Points:**

- Arterial spin labeling (ASL)-MRI enables reliable liver perfusion quantification in healthy individuals.
- ASL-MRI showed great short-term repeatability for liver perfusion measurement in the fasting state.
- ASL-MRI and US showed a moderate correlation in measuring postprandial portal venous hemodynamics change.

**Graphical Abstract:**

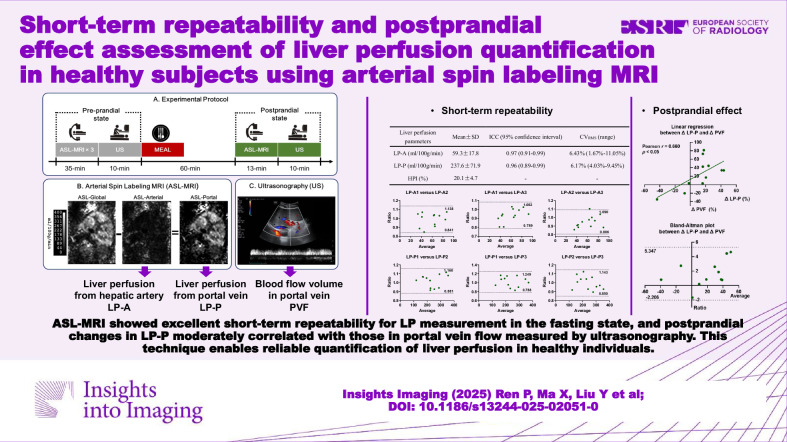

## Introduction

The liver receives its blood supply from two main sources: approximately 75% to 80% via the portal vein (PV), and the remaining portion from the hepatic artery (HA) [1]. Determining relative changes in liver perfusion (LP) may facilitate the earlier detection of primary and metastatic hepatic malignancies as well as liver cirrhosis and other hepatic diseases [2, 3]. Thus, it is essential to quantitatively measure the hepatic arterial and portal venous components of LP. Although Doppler ultrasonography (US) is currently used to quantify blood flow in the PV and HA [4], it necessitates adhering to a precise standard of procedure to avoid significant variability in results [5, 6]. Furthermore, it is unable to measure regional parenchymal flow. Alternatively, dynamic contrast-enhanced computed tomography (CE-CT) and magnetic resonance imaging (CE-MRI) provide both regional and global parameters [7, 8], yet they are, respectively, limited by exposure to X-ray radiation and inadequate spatiotemporal resolution, and the use of contrast agents may pose potential safety risks.

Arterial spin labeling MRI (ASL-MRI) is a non-invasive technique that provides quantitative measurements of microvascular blood flow or perfusion by using labeled protons in blood as an endogenous contrast. This technique has been extensively utilized in brain perfusion imaging [9], and more recently extended to organs outside of the brain [10]. In the field of LP, several studies have demonstrated positive progress using different ASL sequences and labeling strategies [11–14]. For instance, Katada et al reported the portal venous perfusion using a multi-delay pulsed ASL sequence and compared the results with computed tomography perfusion in the same patients [11]. Schalkx et al separately assessed the portal venous and arterial perfusion with pulsed ASL-EPISTAR and pseudo-continuous ASL sequences by labeling the corresponding blood supply [12]. Pan et al employed two separate multi-delay pseudo-continuous ASL acquisitions for the same purpose [13]. Two pseudo-continuous ASL acquisitions were also used by Martirosian et al, and the spatial-temporal LP patterns were investigated [14]. These studies suggest that ASL is a promising method for measuring LP, with pseudo-continuous ASL being the preferred choice for separately assessing portal and arterial perfusion [10]. However, despite these advancements, few studies have investigated the stability of ASL-MRI in human LP quantification, which is crucial before widely applying this technique to LP research and clinical practice.

The aim of this study was to assess the stability of quantifying LP in healthy subjects using the ASL-MRI technique, under both constant and altered perfusion states. First, the short-term repeatability of hepatic arterial and venous perfusion was investigated through three consecutive scans. Subsequently, perfusion state alterations were induced by meal ingestion, and the relative changes in portal venous perfusion were compared with those observed in portal venous blood flow, which were measured using US.

## Materials and methods

### Study design and sample

**Fig. 1:**
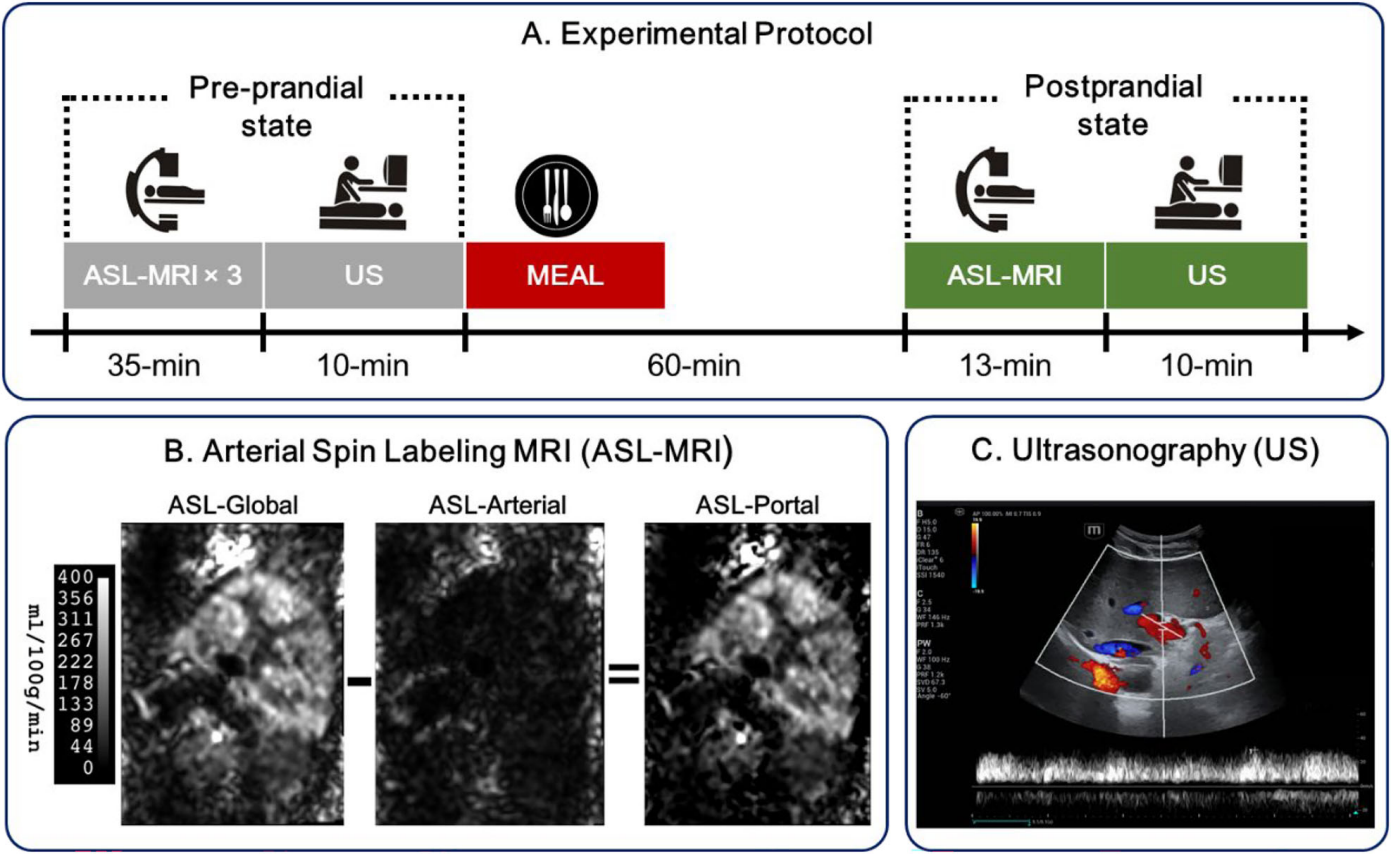
Schematic of the complete experimental protocol (**A**), and typical images of arterial spin labeling MRI (**B**), and Ultrasonography (**C**)

This prospective study received approval from the Ethics Committee of Beijing Friendship Hospital affiliated with Capital Medical University, with the approval number 2024-P2-069. Informed consent was obtained from all participants. Participants were adults (18–65 years) with normal body mass index (BMI 18.5–23.9 kg/m²) and no history of liver disease. Liver diseases such as fatty liver, liver cirrhosis, and tumors were ruled out and confirmed by screening ultrasound and MRI. Additional requirements included no recent medication use (within 2 weeks) and absence of MRI contraindications. Individuals unable to tolerate MRI or with pregnancy/severe comorbidities were excluded. Twelve participants were finally enrolled, and their demographic characteristics of the participants are presented in Table 1. For each subject, the experimental protocol is shown in Fig. 1A. Pre-prandial ASL and ultrasound images were first obtained, and ASL scan was repeated three times consecutively to evaluate the repeatability of this technique in the short term. Then, the subjects consumed a standardized meal providing 600 kcal (55% carbohydrates, 30% fat, and 15% protein), which complied with the Dietary Reference Intakes for China (2023 Edition). Postprandial ASL and ultrasound image acquisition were performed approximately 1 h after the start of meal intake.

**Table 1:** Demographic characteristics of the participants

	Age	Sex (male/female)	Height (m)	Weight (kg)	BMI
Mean ± SD	32.3 ± 7.9	10/2	1.7 ± 0.1	67.3 ± 6.7	22.2 ± 1.2

### MRI protocol

**Fig. 2:**
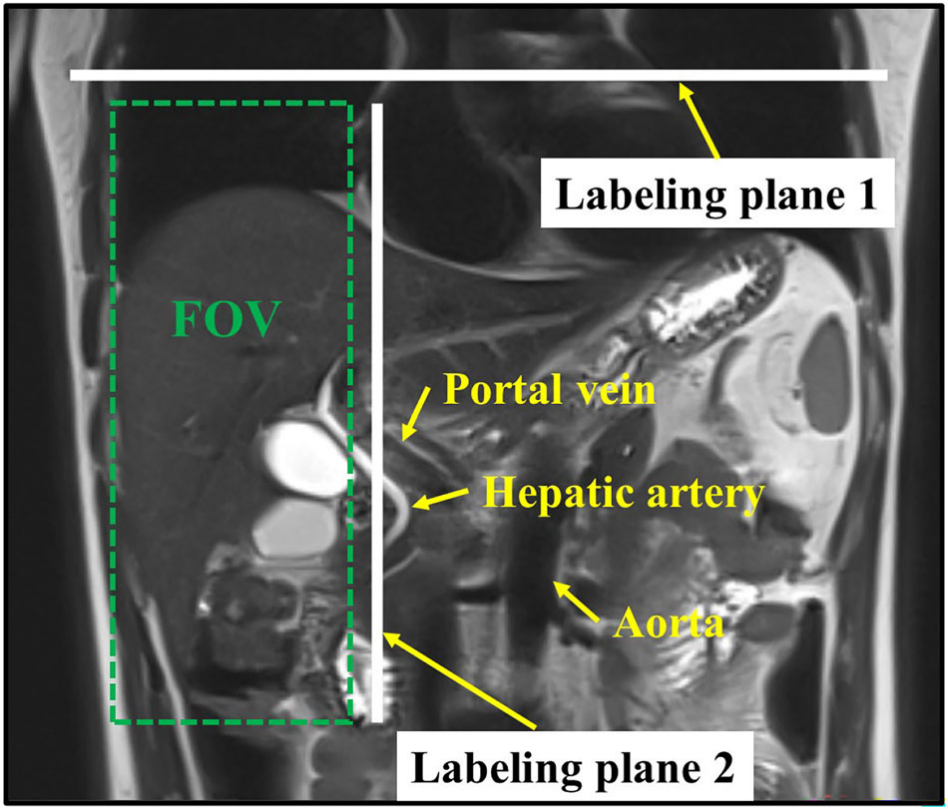
Field of view (FOV) and two labeling planes of ASL-MRI

All ASL-MRI examinations were conducted on a 3.0-Tesla system (MAGNETOM Vida, Siemens Healthineers, Erlangen, Germany) equipped with an eighteen-channel body array coil. For each MRI scan, T2-weighted localizer images in all three planes (coronal, sagittal, and axial) were first collected. After that, two sets of ASL images were acquired by positioning the labeling plane first perpendicular to the aorta and then approximately parallel to the abdominal aorta, as shown in Fig. 2. The hepatic artery perfusion images, represented as ASL-Arterial, and the overall perfusion images, represented as ASL-Global, were captured through these two labeling methods, respectively. The portal vein perfusion images, namely ASL-Portal, were thus obtained by subtracting the ASL-Arterial from ASL-Global, as illustrated in Fig. 1B.

Each set of images was acquired using a three-dimensional turbo gradient spin echo research prototype sequence with a pseudo-continuous ASL scheme, performed in a free-breathing manner. Detailed acquisition parameters, as presented in Table 2, were: repetition time/echo time = 5360/34.6 ms; field of view (FOV) = 187 × 250 mm²; matrix = 96 × 128; slice thickness = 4 mm; 20 slices; labeling duration = 1500 ms; TI = 3000 ms; the longitudinal relaxation time of arterial blood *T*_1,blood_ = 1200 ms; and acquisition time = 5 min 26 s. The perfusion map was estimated on a pixel basis using the following formula:

1 $$\Delta M(t)= \,	2\frac{{M}_{0}}{\lambda }f{T}_{1}^{{\prime} }\alpha \exp \left(\frac{-\Delta t}{{T}_{1,{{\rm{blood}}}}}\right).\exp \left(\frac{-(t-\tau -\Delta t)}{{T}_{1}^{{\prime} }}\right).\left(1-\exp \left(\frac{-\tau }{{T}_{1}^{{\prime} }}\right)\right),\\ \,	{{\rm{with}}}\,\frac{1}{{T}_{1}^{{\prime} }}=\frac{1}{{T}_{1}}+\frac{f}{\lambda }$$

where *f* is the perfusion rate, with a unit of mL/100 g/min; λ denotes the blood-tissue partition coefficient with a value of 0.9 mL/100 g; *M*_0_ is the equilibrium magnetization intensity; α is the inversion efficiency set at 0.98; the arrival time of labeled blood Δ*t* is assumed to be 750 ms; *T*_1,blood_ is; *τ* is the labeling time.

**Table 2:** ASL-MRI acquisition protocol

Parameter	Value/Description
Scanner	3T MAGNETOM Vida (Siemens Healthineers)
Coil	18-channel body array
Sequence	3D turbo gradient spin echo (prototype)
ASL scheme	Pseudo-continuous labeling
Respiratory compensation	Free-breathing acquisition
Labeling positions	1. Perpendicular to aorta 2. Parallel to abdominal aorta
TR/TE	5360/34.6 ms
FOV	187 × 250 mm²
Matrix	96 × 128
Slice thickness	4 mm
Number of slices	20
Labeling duration	1500 ms
Inversion time (TI)	3000 ms
*T*_1,blood_	1200 ms
Acquisition time	5 min 26 s

**Fig. 3:**
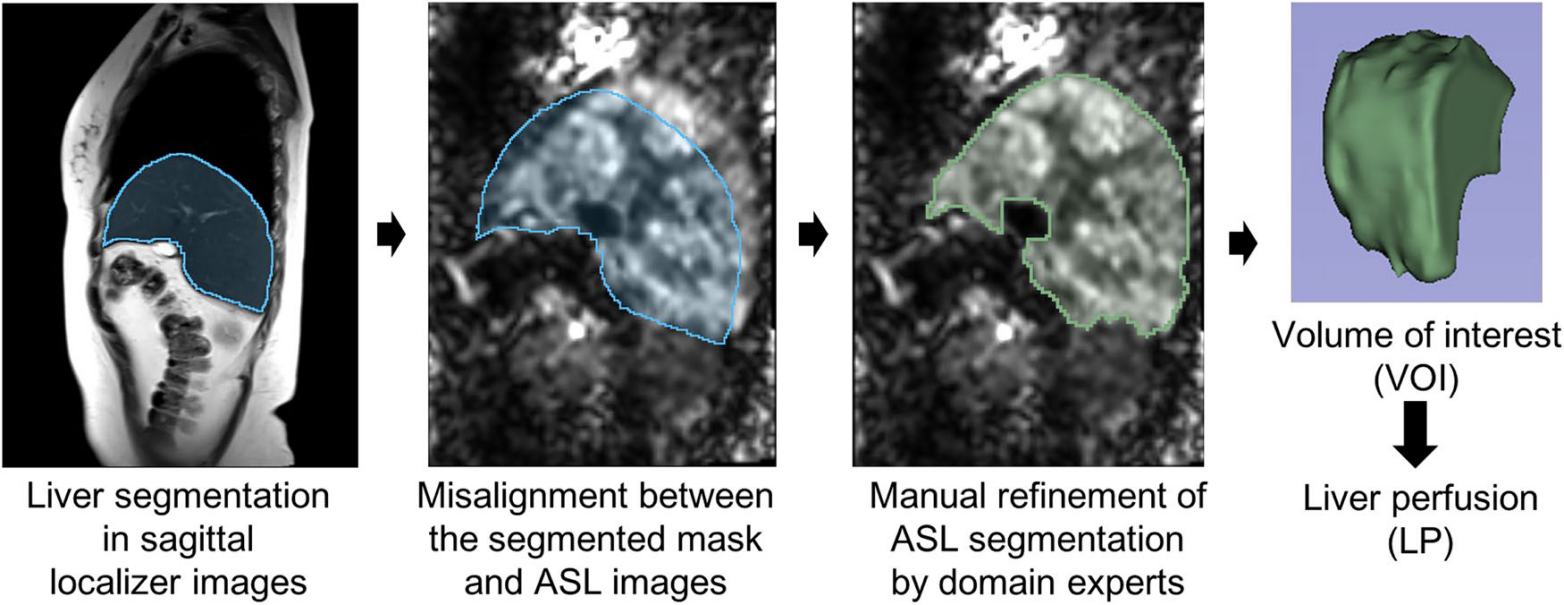
Liver segmentation and liver perfusion calculation process in ASL images

Liver segmentation was performed on the 3D Slicer platform [15]. Given the lower resolution of ASL images compared to structural localizers, a two-stage segmentation protocol was implemented. Initially, automated liver segmentation was conducted on sagittal localizer images using the ‘TotalSegmentator’ plugin [16] to generate baseline anatomical references. Due to respiratory motion discrepancies between breath-hold localization and free-breathing ASL acquisitions, the expert radiologist (P.G.Q., with more than 10 years of experience in abdominal MRI) performed manual slice-wise volumetric region of interest (VOI) adjustments to correct for anatomical misalignment. Final perfusion quantification, excluding prominent blood vessels near the hepatic hilum, was calculated as the mean voxel intensity within the VOI. Figure 3 illustrates this workflow. LP metrics were obtained as follows: LP from HA (LP-A) and LP from PV (LP-P). Additionally, the hepatic perfusion index (HPI) was calculated as a percentage ratio of arterial to global LP:

2 $$HPI=\frac{LP{\mbox{-}}{{\rm{A}}}}{LP{\mbox{-}}A+LP{\mbox{-}}P}\times 100 \%$$

To validate interobserver consistency, 30/47 sets of ASL images were randomly selected and independently analyzed by a second radiologist (F.R.G.) using identical protocols. The results demonstrated excellent agreement (ICCs > 0.98; see Table S1 in the Supplementary Material).

### Ultrasound protocol

B-mode and pulse-wave Doppler US were performed using an ultrasound device (Mindray Resona A20S). The probe frequency was set to 5–10 MHz. All ultrasound scans were performed by an ultrasonographer (X.M.) with 10 years’ experience. All participants were examined in the left lateral position with the transducer in the oblique position in the right upper quadrant of the abdomen. Measurements of the PV diameter (D, cm) and maximum velocity (*V*max, cm/s) were taken at the hilum of the liver just before bifurcation into right and left during suspended inspiration. The diameter was taken by putting the two cursors on the internal wall of the PV. The *V*max was acquired when the insonation angle was less than 60°. A minimum of three portal vein diameters and three *V*max were obtained per subject, and the mean value was calculated. The measurement took about 10 min. D was acquired from the B-mode ultrasound, and *V*max was measured from the Doppler ultrasound. The blood flow volume in PV (PVF, mL/min) was calculated using the following equation [17]:

3 $$PVF=\pi \times {\left(\frac{D}{2}\right)}^{2}\times 0.57{V}_{\max }\times 60$$

### Statistics

Data analysis was performed using SPSS (version 27; IBM). After the Shapiro-Wilk test, the continuous variables all conformed to normal distribution, expressed as mean ± SD. To evaluate the short-term repeatability of ASL-MRI, the pre-prandial LP results were analyzed using the intraclass correlation coefficient (ICC, bidirectional random effects, absolute consistency, single measurement), coefficient of variation (CV), and Bland–Altman analysis.

Two different perfusion conditions were induced through meals to further explore the stability of ASL-MRI. The pre-prandial LP result was determined as the average of the values from three repeated ASL measurements. Paired samples Student’s *t*-test was applied to assess differences in pre-prandial and postprandial PV blood flow data, including variables from both ASL and Doppler measurements. The correlation between the relative change rates of LP-P and PVF was analyzed, and a Bland–Altman plot was constructed to provide information on the consistency distribution between the relative change rates of LP-P and PVF.

ICC values were classified as follows: less than 0.50, poor agreement; 0.50–0.74, moderate agreement; 0.75–0.89, good agreement; and 0.90 or greater, excellent agreement [18]. The Bland–Altman method was used to analyze the mean ratio in LP between measurements. Ratios within the 95% limits of agreement (mean ± 1.96 SD) were considered to have high agreement. *p* < 0.05 was considered to indicate a statistically significant difference or correlation.

## Results

### Experiments

All ASL and ultrasound experiments were successfully performed. Breathing instructions were successfully followed by all subjects in the ASL measurement. A typical example of ASL and Doppler images is shown in Fig. 1B, C. Postprandial ASL imaging was performed 60 ± 5 min after the start of meal ingestion, with a minimum of 54 and a maximum of 69 min. Due to personal reasons, one of the subjects declined to undergo the postprandial examination. Consequently, the pre-meal reproducibility analysis comprised 12 data points, while the combined pre-meal and post-meal analysis encompassed only 11 data points. Comprehensive measurements from all participants, including pre- and postprandial MRI and ultrasound results, can be found in Table S2 in the Supplementary Material.

### Short-term repeatability

In a fasting state, the LP values (in mL/100 g/min) of all participants obtained from three measurements were LP-A = 59.3 ± 17.8 and LP-P = 237.6 ± 71.9, and the HPI values were 20.1 ± 4.7%, as shown in Table 3. The ICC values of LP-A and LP-P were 0.97 and 0.96, respectively, which were greater than 0.90, indicating excellent agreement between different measurements. The root mean square values of CV (CV_RMS_) of all subjects were 6.43% for LP-A and 6.17% for LP-P, both at a relatively low level below 10%, indicating great repeatability of the perfusion results.

**Table 3:** Liver perfusion and hepatic perfusion index values in three measurements, and the results of intraclass correlation coefficient and coefficient of variation analysis (*N* = 12)

Liver perfusion parameters	Mean ± SD	ICC (95% confidence interval)	CV_RMS_ (range)
LP-A (mL/100 g/min)	59.3 ± 17.8	0.97 (0.91–0.99)	6.43% (1.67%–11.05%)
LP-P (mL/100 g/min)	237.6 ± 71.9	0.96 (0.89–0.99)	6.17% (4.03%–9.45%)
HPI (%)	20.1 ± 4.7	-	-

*LP-A* liver perfusion from hepatic artery, *LP-P* liver perfusion from portal vein, *HPI* hepatic perfusion index, *ICC* intraclass correlation coefficient, *CV*_RMS_ root mean square values of coefficient of variation

**Fig. 4:**
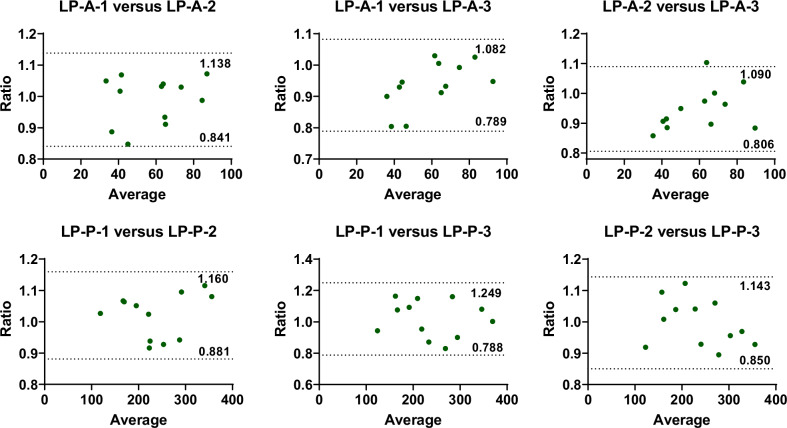
Bland–Altman plots for analyzing the consistency of liver perfusion results between different measurements (*N* = 12). LP-A, liver perfusion from hepatic artery; LP-P, liver perfusion from portal vein; -i (*i* = 1, 2, 3) means results obtained from the i*-*th ASL measurement

In Fig. 4, the Bland–Altman plots between every two pairs in the three measurements also indicated a high degree of consistency in the LP results, with almost all subjects’ data falling within the 95% consistency limit, except for one subject in the Bland–Altman plot comparing the second and the third LP-A, whose data fell outside the consistency interval.

### Postprandial effect

**Fig. 5:**
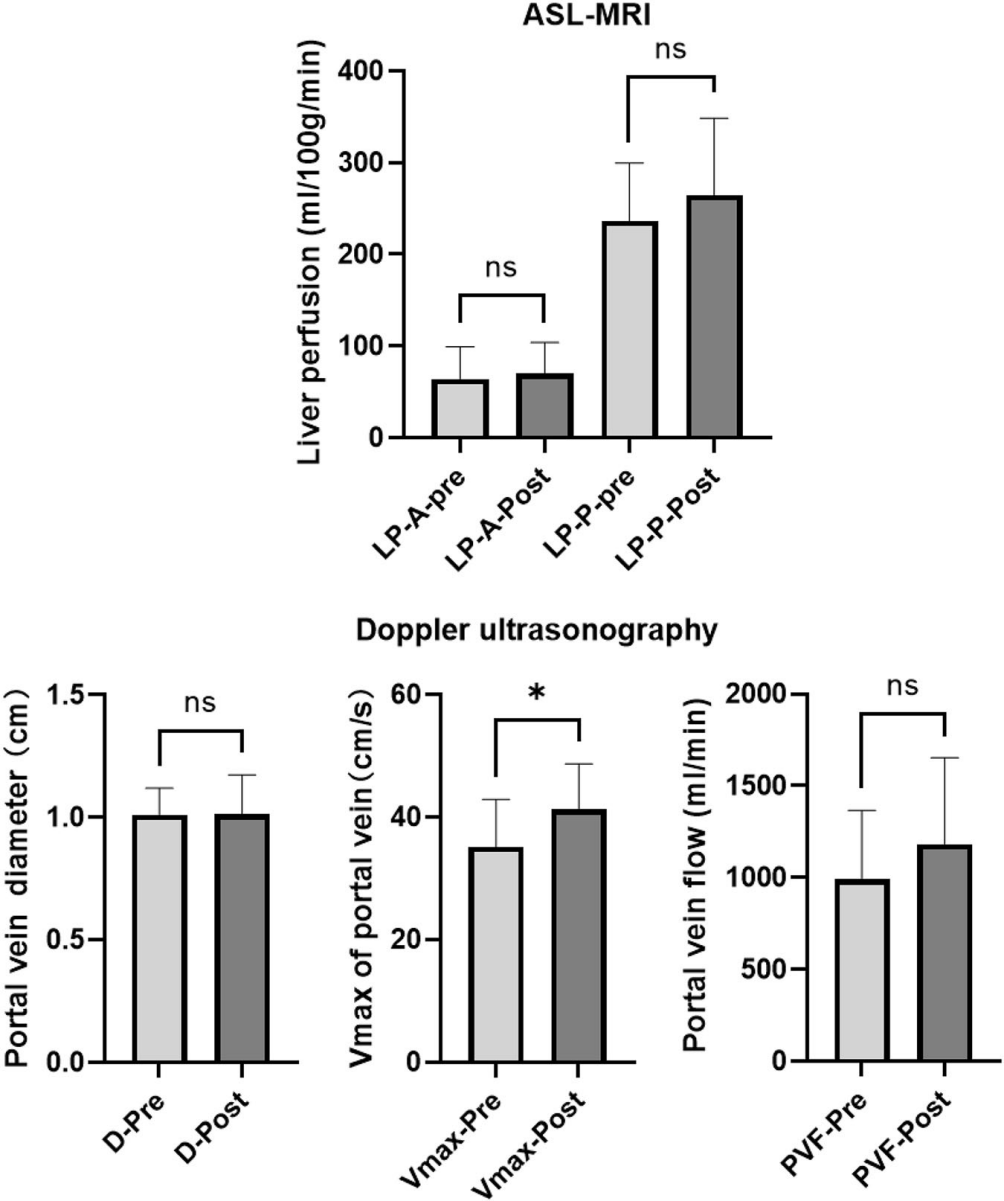
Pre- and postprandial liver blood flow parameters measured by ASL-MRI and Doppler ultrasonography (*N* = 11). LP-A, liver perfusion from hepatic artery; LP-P, liver perfusion from portal vein; PVF, blood flow volume in portal vein; -pre and -post, respectively, mean the pre-prandial and the postprandial state

Compared to the fasting state, there were no significant changes in LP-A and LP-P measured by ASL after meals. For the US examinations, PV diameter and PVF showed no significant variation, while the maximum velocity of blood flow increased significantly (*p* < 0.05). The results are presented in Table 4 and Fig. 5, which indicate that there are significant individual differences in the changes of liver blood flow after meals.

**Table 4:** Changes in liver perfusion measured by ASL-MRI and portal vein flow measured by Doppler ultrasonography before and after food ingestion (*N* = 11)

	Pre-prandial		Postprandial		Pre-Post	*t*-test
	Mean ± SD	(min, max)	Mean ± SD	(min, max)	Mean ± SD	
ASL, values are given in mL/100 g/min.						
LP-A	56.5 ± 16.1	(34.9, 83.7)	64.9 ± 28.0	(36.3, 115.1)	−8.5 ± 18.4	*p* = 0.16
LP-P	236.6 ± 62.9	(158.5, 388.0)	264.4 ± 84.0	(150.3, 450.1)	−27.8 ± 57.2	*p* = 0.14
Doppler ultrasonography, values are given in mL/min.						
PVF	991.9 ± 373.5	(452.4, 1753.4)	1204.9 ± 471.9	(534.8, 1927.3)	−213.0 ± 401.4	*p* = 0.11

*LP-A* liver perfusion from hepatic artery, *LP-P* liver perfusion from portal vein, *PVF* blood flow volume in the portal vein

**Fig. 6:**
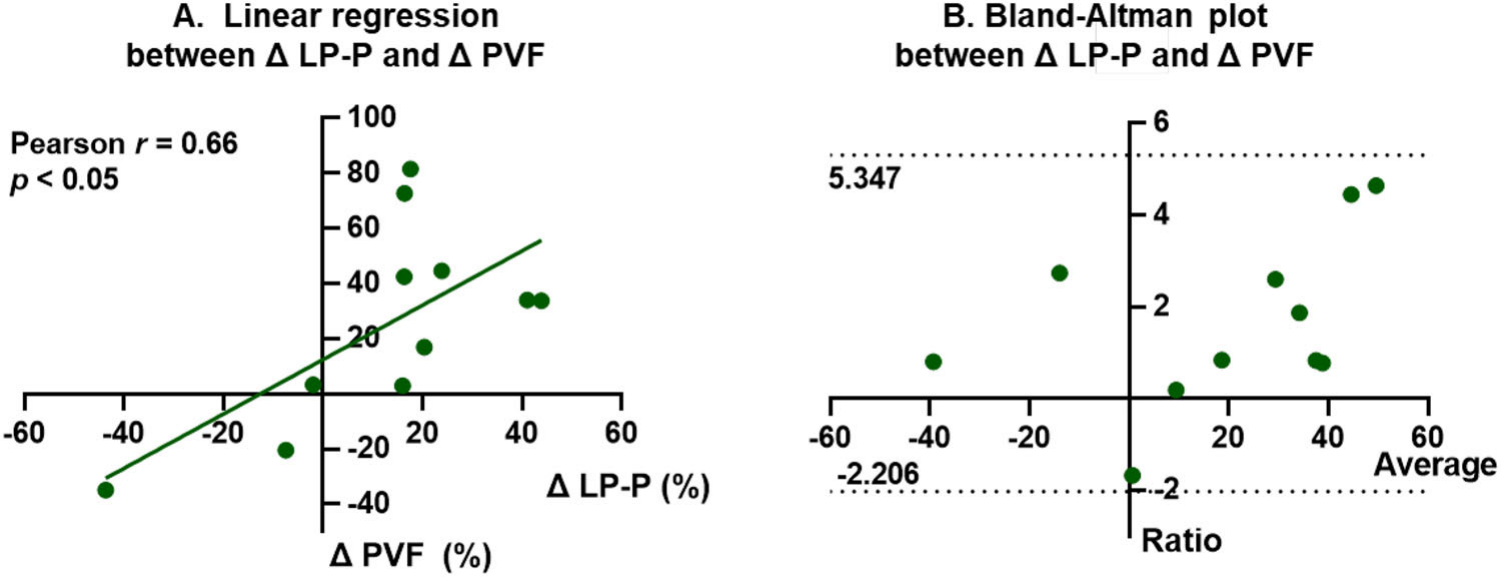
The linear regression (**A**) and Bland–Altman plot (**B**) between the relative postprandial change of LP-P measured by ASL and that of PVF measured by Doppler ultrasonography (*N* = 11). LP-P, liver perfusion from the portal vein; PVF, blood flow volume in the portal vein

The consistency of ASL and Doppler US in measuring changes in liver blood flow before and after meals was analyzed, as shown in Fig. 6. The relative change rate of LP-P exhibited a moderate positive correlation with that of PVF, with a Pearson correlation coefficient *r* = 0.66. The Bland–Altman plot showed that all data points for the 11 subjects fall within the limits of agreement, indicating good consistency between the two techniques in measuring liver blood flow changes.

## Discussion

This study demonstrated the stability of quantifying hepatic arterial and portal venous LP in healthy subjects using ASL-MRI, in both constant and meal-induced variable perfusion states. ASL-MRI exhibited excellent short-term repeatability for LP measurement in the fasting state, with low variability between consecutive scans. Furthermore, while there were no significant changes in LP measured by ASL-MRI after meals, this technique showed moderate correlation and good consistency with US in detecting changes in PVF.

A pseudo-continuous ASL sequence was employed, with blood labeling planes positioned first vertically and then parallel to the aorta for two separate acquisitions. This allowed for the generation of arterial and global LP images, from which the portal venous perfusion image was derived by subtracting the former from the latter. Notably, the portal venous perfusion image was obtained indirectly, differing from methods reported in existing literature. In previous research, portal venous perfusion images were typically acquired through a single labeling and acquisition process, where the labeling plane was usually positioned below the hepatic artery, intersecting the superior and inferior mesenteric and splenic veins [14], or approximately perpendicular to the PV but also intersecting the hepatic artery and descending aorta [13]. In contrast, the labeling strategy adopted in this study is easier to implement and free from the interference of incomplete “double inversion” [13], thereby providing a novel labeling strategy for ASL-based portal venous perfusion measurement.

Both the arterial and portal venous LP results exhibited excellent short-term reproducibility. Notably, the short-term repeatability of LP-A was slightly inferior to that of LP-P, aligning with the results obtained using very low-dose hepatic perfusion CT [19]. This is directly related to the smaller absolute values of LP-A, which are prone to measurement errors. Additionally, the ASL signal is directly proportional to blood flow, and given that arterial perfusion is much lower than PV perfusion, the resulting measurements may exhibit increased variability.

The LP obtained with ASL in healthy volunteers under the fasting condition was 59.3 ± 17.8 for LP-A and 237.6 ± 71.9 for LP-P, with the perfusion unit being mL/100 g/min for both here and in the following text. Our arterial and portal venous LP values are higher than most of those reported in the published literature, despite that the LP values differed between studies and showed large standard deviations [10, 12]. Taking LP-P as an example, the average values reported in the literature for healthy subjects using ASL range from 63 to 254 [11–14]; the values measured using other modalities are 102 ± 35 (*N* = 24, CE-CT) [20] and 126.3 ± 66.7 (*N* = 10, CE-MRI) [21]. Portal perfusion measured in our study is comparable to that reported by Katada et al, which was 254.3 ± 58.3 (*N* = 5) [11]. These discrepancies are attributed to multiple factors such as imaging techniques, parameter settings, post-processing methods, as well as individual differences and states of the subjects. Despite variations in absolute values of LP, the proportion of arterial perfusion to total perfusion, known as HPI, falls within the range reported in the published literature. Our study found an average HPI of 20.1% with a range of 12.8% to 26.5% among twelve healthy subjects. Abdullah et al reported an average of 30% with a range of 10% to 50% (*N* = 13) [22], while Martirosian et al reported an average of 26% with a range of 6% to 39% (calculated, *N* = 8) [23].

The reliability of ASL-MRI was further assessed by comparing fasting-to-postprandial hemodynamic changes. The 1-h interval was chosen to capture significant and relatively stabilized hemodynamic alterations based on the reported evidence: Dauzat et al documented sustained postprandial hemodynamic changes, peaking at 30 min and plateauing by 60 min [24], Gaiani et al confirmed significant flow augmentation at this timepoint [25], and Obrzut et al observed liver stiffness changes lasting until 2.5 h post-meal via MR elastography, with peaks also occurring at 30 min [26]. In this study, we found no statistically significant change in LP-A measured by ASL-MRI after a meal in 11 healthy subjects. This finding aligns with the observations reported in the study of Schalkx et al [12]. When examining hemodynamic changes relating to the PV postprandially, ASL measurements showed an increase in LP-P for eight out of eleven subjects, although the LP-P changes among all subjects did not reach statistical significance. Doppler US of the PV demonstrated a significant increase in maximum velocity after eating (*p* < 0.05), and the changes in PVF mirrored those of LP-P, with nine subjects exhibiting an increase. The postprandial increase in portal venous blood flow was consistent with previous studies using MRI [12, 27] and Doppler ultrasound [24, 25]. The decrease in LP-P observed in a minority of subjects may be attributed to the variability in individual responses to food intake. While our measurement window was empirically chosen based on established hemodynamic evolution patterns, it may not capture individual peak perfusion changes. According to the literature, 30 min is considered the timepoint with the most significant postprandial blood flow changes [25, 26]. In future ASL-MRI examinations, meal and fasting time should be considered and incorporated into the standardization process for LP quantification.

Doppler US is a widely used clinical technique for measuring blood flow in blood vessels. Our study compared PVF measured using this technique with LP-P assessed by ASL-MRI, evaluating the consistency between the two methods in the context of postprandial hepatic hemodynamic changes after a meal. A comparison was made between their relative changes before and after meals, and the results demonstrated a moderate correlation (*r* = 0.66) and excellent consistency. The moderate (rather than strong) correlation between PVF and LP-P likely stems from their divergences in physiological capture domains and technical measurement principles. While both metrics reflect the hemodynamic situation of the PV, their physiological underpinnings differ: PVF captures direct blood flow of the PV, whereas LP-P reflects complex microvascular perfusion modulated by hepatic compliance and autoregulation. Technically, PVF is influenced by vascular cross-sectional area, probe angle, and segmental variations in Doppler measurements. In contrast, LP-P represents the downstream hepatic tissue perfusion derived from ASL-MRI, which depends on factors such as arterial spin labeling strategy, regional perfusion heterogeneity, and post-processing algorithms. While the above variations exist, the observed consistency in directional changes across 8/11 subjects suggests the potential of ASL-MRI’s clinical validity for quantifying meal-induced perfusion change.

This study has several limitations. First, the study employed manual post-processing for the ASL images, and future efforts should focus on overcoming the poor signal-to-noise ratio of ASL images to achieve automatic liver segmentation and perfusion quantification. Second, given a greater emphasis on the stability of the technique itself, we have only tested the repeatability of ASL-MRI over a short period of time. Further research is required to assess its repeatability in LP measurements across multiple visits, i.e., over a longer duration. Finally, the modest sample size represents a key limitation of this study. While the primary focus was to demonstrate methodological feasibility, the limited cohort likely reduced statistical power to detect postprandial hemodynamic changes and to resolve inter-individual variability in meal-induced portal-hepatic responses. This constraint may also affect the generalizability of our findings. Future studies with larger cohorts are warranted to (1) validate the reproducibility of ASL-derived perfusion metrics across diverse populations, (2) disentangle biological variability from technical limitations, and (3) clarify dynamic interactions between portal flow and downstream LP under physiological perturbations such as feeding.

## Conclusions

In conclusion, this study demonstrates that arterial spin labeling MRI enables reliable quantification of LP in healthy individuals. ASL-MRI exhibited great short-term repeatability for arterial and portal venous LP measurement in the fasting state, and the changes in portal venous perfusion observed between the fasting and postprandial states are consistent with those of PV flow measured by Doppler US. These findings highlight the potential of ASL-MRI as a non-invasive and reliable imaging modality, offering new insights into liver hemodynamics.

## Supplementary information

ELECTRONIC SUPPLEMENTARY MATERIAL

## Data Availability

The data that support the findings of this study are available on request from the corresponding author, [Rui Wang], upon reasonable request.

## Abbreviations

ASL-MRI: Arterial spin labeling magnetic resonance imaging
CE-CT: Contrast-enhanced computed tomography
CE-MRI: Contrast-enhanced magnetic resonance imaging
CV: Coefficient of variation
D: Diameter of portal vein
HA: Hepatic artery
HPI: Hepatic perfusion index
ICC: Intraclass correlation coefficient
LP: Liver perfusion
LP-A: Liver perfusion from hepatic artery
LP-P: Liver perfusion from portal vein
PV: Portal vein
PVF: Blood flow volume in portal vein
SD: Standard deviation
US: Ultrasonography
Vmax: Maximum velocity of portal vein
VOI: Volume of interest
