# Supplementary material for: Short-term repeatability and postprandial effect assessment of liver perfusion quantification in healthy subjects using arterial spin labeling MRI
Source: Insights Imaging. 2025 Aug 5;16:167. doi: 10.1186/s13244-025-02051-0 (PMC12325835; doi:10.1186/s13244-025-02051-0)
Supplement: Supplementary file 1 — ELECTRONIC SUPPLEMENTARY MATERIAL [file 13244_2025_2051_MOESM1_ESM.pdf]

Short-term repeatability and postprandial effect assessment of liver perfusion quantification in healthy subjects using arterial spin labeling MRI

ELECTRONIC SUPPLEMENTARY MATERIAL

Table S1. Interobserver reliability analysis of liver perfusion parameters in 30 randomly selected measurements using semi-automated VOI delineation

Liver perfusion parameters	Observer 1	Observer 2	ICC (95% confidence interval)	P values
LP-A (ml/100g/min)	260.4±67.7	258.1±65.9	0.982(0.963, 0.992)	<0.0001
LP-P (ml/100g/min)	63.9±19.5	64.7±19.7	0.984(0.966, 0.992)	<0.0001
HPI (%)	18.7±7.7	19.1±8.2	0.982(0.963, 0.991)	<0.0001

LP-A liver perfusion from hepatic artery, LP-P liver perfusion from portal vein, HPI hepatic perfusion index, ICC: intraclass correlation coefficient (bidirectional random effects, absolute consistency, single measurement), Observer 1: PG.Q. (primary radiologist), Observer 2: FR.G. (validation radiologist).

Table S2. Pre- and postprandial liver perfusion (ASL-MRI) and portal venous hemodynamics (US) results in 12 subjects

Subject number {age, gender, BMI}	ASL-MRI						**US**					
	LP-A (ml/100g/min)		LP-P (ml/100g/min)		HPI (%)		D (cm)		Vmax (cm/s)		PVF (mL/min)	
	Pre*	Post	Pre*	Post	Pre*	Post	Pre	Post	Pre	Post	Pre	Post
Subject 1 {36, M, 21.3}	65.8	66.2	199.0	246.5	24.8	21.2	1.1	1.3	41.8	44.5	1332.7	1927.2
Subject 2 {38, M, 23.4}	63.1	53.1	266.7	150.3	19.1	26.1	1.2	1.0	46.0	44.1	1753.4	1141.5
Subject 3 {42, M, 22.2}	47.2	38.6	158.5	155.4	22.9	19.9	1.0	1.1	46.0	37.6	1256.0	1297.7
Subject 4 {42, M, 22.5}	38.5	46.5	223.2	262.4	14.7	15.1	1.0	1.1	31.3	53.7	911.0	1654.2
Subject 5 {41, M, 23.9}	42.0	39.8	163.1	229.9	20.5	14.8	1.0	1.1	34.7	43.0	954.8	1279.2
Subject 6 {32, F, 21.7}	34.9	36.3	237.7	219.9	12.8	14.2	1.0	0.8	25.0	31.1	672.1	534.8
Subject 7 {24, M, 20.5}	83.7	88.5	388.0	450.1	17.7	16.4	1.1	1.0	24.3	27.4	730.3	751.7
Subject 8 {35, F, 21.5}	42.9	52.0	273.8	318.5	13.5	14.0	0.8	0.8	28.0	36.9	452.4	644.5
Subject 9 {22, M, 22.6}	66.0	69.1	257.4	299.5	20.4	18.7	1.0	1.2	38.9	44.9	1048.0	1810.6
Subject 10 {21, M, 20.3}	63.4	109.2	224.1	322.3	22.0	25.3	0.9	0.8	31.7	47.0	636.1	850.9
Subject 11 {24, M, 22.5}	73.9	115.1	210.8	253.7	26.0	31.2	1.1	1.0	38.9	43.9	1164.0	1361.4
Subject 12 {30, M, 23.7}	89.7	-	248.6	-	26.5	-	1.0	-	35.9	-	915.3	-

LP-A liver perfusion from hepatic artery, LP-P liver perfusion from portal vein, HPI hepatic perfusion index, D diameter of portal vein, Vmax maximum velocity of portal vein, PVF blood flow volume calculated from D and Vmax, * average values calculated from three pre-prandial measurements. - missing data (Subject 12 declined postprandial protoco

Insights Imaging (2025) Ren P, Ma X, Liu Y, et al.
